# Health‐related quality of life among patients with esophageal, gastric, and colorectal cancer at Kenyatta National Hospital

**DOI:** 10.1002/cnr2.2038

**Published:** 2024-03-20

**Authors:** Amsalu Degu, Peter N. Karimi, Sylvia A. Opanga, David G. Nyamu

**Affiliations:** ^1^ United States International University–Africa, Department of Pharmaceutics and Pharmacy Practice School of Pharmacy and Health Sciences Nairobi Kenya; ^2^ University of Nairobi, Department of Pharmacology Clinical Pharmacy and Pharmacy Practices, Faculty of Health Sciences Nairobi Kenya

**Keywords:** determinants, gastrointestinal cancers, health‐related quality of life, Kenyatta National Hospital

## Abstract

**Background:**

Despite the advancement of modern treatment approaches, several studies indicated a diminished health‐related quality of life (HRQoL) in patients with gastrointestinal cancer. However, there is insufficient data about the HRQoL of gastrointestinal cancer patients in Kenya.

**Aims:**

The study aimed to investigate HRQoL and its determinants in gastrointestinal cancer patients at Kenyatta National Hospital.

**Methods:**

A cross‐sectional study was employed among 160 esophageal, 103 gastric, and 96 colorectal cancer patients. The patient list, identified by unique hospital identification numbers, was obtained from records. Eligibility was assessed based on predetermined criteria, and the hospital identification numbers were reshuffled. Study participants were then randomly selected daily during the data collection period. Data were collected using a researcher‐administered European Organization for Research and Treatment of Cancer quality of life questionnaire. The data entry and analysis were carried out using Statistical Package for the Social Sciences 26.0 statistical software. A bivariate and multivariate binary logistic regression analysis was employed to investigate determinants of HRQoL at a 0.05 level of significance.

**Results:**

Most esophageal (*N* = 118, 73.7%), gastric (*N* = 75, 72.8%), and colorectal (*N* = 72, 75%) cancer patients had poor overall HRQoL. In the social (*p* = .04) and cognitive (*p* = .02) domain of HRQoL, esophageal cancer patients had a significantly lower mean score as compared to gastric cancer patients. Colorectal cancer patients had the highest mean score in physical functioning (*p* = .01) as compared with gastric cancer patients. Nonetheless, gastric cancer patients had the highest mean score in emotional functioning domains of quality of life as compared to esophageal (*p* = .04) and colorectal (*p* < .001) cancer patients The study revealed a low mean HRQoL score in the majority of the symptom domains of quality of life. A statistically significant difference in all domains of HRQoL was not observed in various treatment modalities of gastrointestinal cancer. Advanced‐stage (stages III and IV) and co‐morbidities were significant determinants of poor HRQoL.

**Conclusions:**

The overall HRQoL of gastrointestinal cancer patients was poor. Advanced‐stage cancer and co‐morbidities were significant determinants of poor HRQoL. Therefore, intensification of routine monitoring of the disease and the treatments should be actively implemented to improve the HRQoL.

## 1 BACKGROUND

The global burden of cancer has a substantial physical, emotional, and financial stress on individuals, families, communities, and healthcare systems. This burden of cancer has a tremendous negative impact on low and middle‐income countries due to ill‐equipped healthcare systems.^1^ Gastrointestinal cancer accounted for 26% of the global cancer incidence and 35% of all cancer‐related deaths worldwide.^2^ By 2030, it is anticipated that gastrointestinal cancer will surge by 73%, with over 90% of these cancer cases being diagnosed at an advanced stage in sub‐Saharan Africa.^3^ These late presentations can compromise the health‐related quality of life (HRQoL) of patients due to the advancement of the disease.

From diagnosis to treatment, cancer survivors face mental, physical, and economic challenges and confusion regarding their social roles.^4^ Numerous studies have demonstrated that cancer treatment adversely affects the HRQoL of patients.^5^, ^6^, ^7^, ^8^, ^9^, ^10^, ^11^ In developing countries, HRQoL is generally low among cancer patients.^12^, ^13^ A recent systematic review showed that most cancer patients had a suboptimal overall HRQoL in Sub‐Saharan Africa.^14^ In addition, a major reduction in HRQoL is observed as cancer progresses, with a sharp decline in the advanced stages.^15^, ^16^, ^17^, ^18^

The majority of studies indicated a diminished overall HRQoL across various domains in patients with gastrointestinal cancer following treatment.^10^, ^19^, ^20^, ^21^ There is a significant scarcity of studies conducted in African settings, including Kenya, that investigate the HRQoL in patients with gastrointestinal cancer. Moreover, the majority of studies failed to examine different domains of HRQoL and HRQoL disparities based on various treatment modalities. Therefore, the present study aimed to investigate the HRQoL of patients with gastrointestinal cancer at Kenyatta National Hospital in Kenya.

## 2 METHODS

### 2.1 Study design, target population, study setting, and period

A cross‐sectional study was employed at the Department of Oncology among hospitalized and ambulatory adult esophageal, gastric, and colorectal cancer patients. This study design was employed to assess HRQoL and its determinants at a single point in time. The study was conducted at Kenyatta National Hospital, the largest referral and teaching facility in Kenya which is located in Upper Hill Nairobi County. It was established in 1901 during the British colonial administration. The hospital offers a wide range of medical services, including specialized care such as cancer treatment. Since it is the largest referral facility, the hospital serves a diverse population from various regions across Kenya. That is why the facility was selected to conduct the present study. The research was carried out from June 1 to December 31, 2022.

### 2.2 Inclusion and exclusion criteria

All adult patients (18 years and above) with gastric cancer, esophageal cancer, and colorectal cancer treated in the hospital and who signed informed consent were included in the study. In addition, the patients were required to complete at least one treatment modality and had documentation about their treatment regimens, stage of cancer and histological type to be involved in the study. Unconscious, unwilling, and below 18‐year‐old patients were excluded from the study. Further, patients who did not complete at least one treatment specific to their cancer and had incomplete data about their treatment regimens were also excluded from the study.

### 2.3 Sample size determination and sampling techniques

Single population proportion formula was used to compute the sample size for all three gastrointestinal cancers.^22^ Hence, the final sample size with a 10% adjustment for non‐response rate comprised 160 esophageal, 103 gastric, and 96 colorectal cancer patients. The list of patients in active treatment was sourced from the Department of Health Information. The research assistants examined the medical records for eligibility in accordance with the study's eligibility criteria. The list of hospitalized and ambulatory esophageal, gastric, and colorectal cancer patients was sourced from the records using their unique hospital identification numbers. The research assistants examined the records of the patients to determine their suitability for inclusion using the study's specified eligibility criteria. After swapping the hospital identifying numbers, the study participants were randomly selected daily by a lottery method.

### 2.4 Data collection instruments and techniques

The European Organization for Research and Treatment of Cancer quality of life questionnaire (EORTC QLQ‐30), EORTC QLQ–OES18 (esophageal cancer module), EORTC QLQ–STO22 (gastric cancer module), and EORTC QLQ–CR29 (colorectal module) were employed to assess the HRQoL.^23^, ^24^, ^25^, ^26^, ^27^ The general and the cancer‐specific HRQoL tools were employed to comprehensively assess the quality of life in gastrointestinal patients. The EORTC QLQ‐30 tool employed in the current study was validated to be used among cancer patients in Kenya.^28^ After training about the optimal use of data collection tools, the principal investigator and research assistants were involved in the data collection. After randomly selecting the study participants using the lottery method with their hospital identification numbers, the research assistants explained the study's objectives to the recruited study participants. The data were collected by interviewing the patients using the standard HRQoL questionnaire after getting written informed consent from the study participants.

### 2.5 Data analysis

The data entry and analysis were conducted using Statistical Package for the Social Sciences (SPSS) version 26.0 software. The mean and standard error of the mean were used to report the mean age and mean score of HRQoL of the patients under the different domains of quality of life. Patients with a high mean score (≥60) on a global scale and the functional domains and a low mean score (<60) on the symptom domains of quality of life were considered to have good HRQoL. Poor HRQoL was represented by a low mean score (<60) on the global and functional scale and a high mean score (≥60) on the symptom scale of quality of life.^29^

Frequency and percentage were used to report the sociodemographics, clinical characteristics, treatment regimens, and overall HRQoL of the study participants. Bivariate and multivariate binary logistic regression analysis was employed to investigate the determinants of HRQoL. The determinants of HRQoL were reported using crude and adjusted odds ratios. An independent variable with a *p*‐value of ≤.05 in multivariate binary logistic regression was considered a statistically significant determinant of HRQoL. A one‐way analysis of variance (ANOVA) post hoc analysis was conducted to examine the mean score difference based on diagnosis and treatment modalities. The baseline group equivalency of categorical was assessed using the chi‐square test while one‐way ANOVA was employed for continuous variables.

## 3 RESULTS

### 3.1 Descriptions of the sample

The predominant portion of gastrointestinal cancer patients were males and above 60 years old. Esophageal, gastric, and colorectal cancer patients had mean ages of 60.5 ± 12.7, 59.8 ± 1.3, and 53 ± 1.5 years, respectively. The mean age was statistically different between esophageal and colorectal (*p* < .001) and gastric and colorectal cancer patients (*p* = .001). Nonetheless, the mean age difference between esophageal and gastric cancer was not significant (*p* = .9). In the present study, there was a significant difference in histological types (*p* < .001), stage of cancer (*p* < 0.001), co‐morbidity (*p* = .014) and treatment regimens (*p* < .001) among gastrointestinal cancer patients.

The majority of gastric (*N* = 101, 98.1%) and colorectal (*N* = 95, 99.1%) cancer cases were adenocarcinomas, whereas most esophageal cancer patients (*N* = 145, 90.6%) had squamous cell carcinoma. Most esophageal cancer patients had Stages II (*N* = 55, 34.4%) and III (*N* = 53, 33.1%) disease at diagnosis while most gastric patients had Stage III (*N* = 46, 44.7%) and Stage IV (*N* = 35, 33.9%) diseases at the time of diagnosis. Likewise, the majority of colorectal cancer patients had also Stages III (*N* = 32, 33.3%) and IV (*N* = 50, 52.1%) diseases. Most esophageal (*N* = 89, 55.6%) and gastric (*N* = 65, 63.1%) cancer patients had co‐existing co‐morbid conditions at diagnosis. In contrast, only 42.5% (*N* = 41) of colorectal cancer patients had co‐morbidities (Table 1).

**TABLE 1:** Socio‐demographic, clinical characteristics, and treatment regimens of gastrointestinal cancer patients.

	Esophageal cancer (*n* = 160)	Gastric cancer (*n* = 103)	Colorectal cancer (*n* = 96)	
Variable	Frequency, *N* (%)	Frequency, *N* (%)	Frequency, *N* (%)	*p*‐value
Age (in years)				<.001*
<60 years	71 (44.4)	47 (45.6)	66 (68.8)	
≥60 years	89 (55.6)	56 (54.4)	30 (31.2)	
Gender				.819
Male	97 (60.6)	64 (62.1)	62 (64.6)	
Female	63 (39.4)	39 (37.9)	34 (35.4)	
Histological type				<.001*
Adenocarcinoma	15 (9.4)	101 (98.1)	95 (99)	
Squamous cell carcinoma	145 (90.6)	2 (1.9)	1 (1)	
Stage of cancer				<.001*
Stage I	11 (6.9)	1 (1.0)	3 (3.1)	
Stage II	55 (34.4)	21 (20.4)	11 (11.5)	
Stage III	53 (33.1)	46 (44.7)	32 (33.3)	
Stage IV	41 (25.6)	35 (33.9)	50 (52.1)	
Comorbidity				.014*
Present	89 (55.6)	65 (63.1)	41 (42.7)	
Absent	71 (44.4)	38 (36.9)	55 (57.3)	
Treatment regimen				<.001*
Surgery	49 (30.6)	23 (22.3)	12 (12.5)	
Chemotherapy and radiotherapy	32 (20)	6 (5.8)	16 (16.7)	
Chemotherapy	6 (3.8)	36 (35)	21 (21.9)	
Radiotherapy	17 (10.6)	1 (1)	1 (1)	
Surgery and chemotherapy	4 (2.5)	13 (12.6)	25 (26)	
Symptomatic management	30 (18.8)	20 (19.4)	6 (6.3)	
Radiotherapy, chemotherapy and surgery	11 (6.9)	4 (3.9)	15 (15.6)	
Radiotherapy and surgery	11 (6.9)	0 (0)	0 (0)	

* Statistically significant at *p* < .05.

Surgery (*N* = 49, 30.6%) and chemotherapy and radiotherapy combination (*N* = 32, 20.0%) were the most frequent treatment modalities used among esophageal cancer patients. Chemotherapy (*N* = 36, 35.0%) and surgery (*N* = 23, 22.3%) were the most frequently prescribed treatment modalities for patients with gastric cancer. A quarter of colorectal cancer patients (*N* = 25, 26.0%) were treated with surgery and chemotherapy. The other 74% (*N* = 70) of colorectal cancer patients were treated with chemotherapy alone (*N* = 21, 21.9%), surgery alone (*N* = 12, 12.5%), radiotherapy alone (*N* = 1, 1%), chemotherapy and radiotherapy combination (*N* = 16, 16.7%), radiotherapy, chemotherapy and surgery combination (*N* = 15, 15.6%) and symptomatic management (*N* = 6, 6.3%) modalities (Table 1).

### 3.2 HRQoL according to the diagnosis

Concerning esophageal cancer patients, most of them (*N* = 118, 73.7%) had a poor (score on the global scale <60) overall HRQoL. About one‐fourth (*N* = 42, 26.3%) had a good (score on the global scale ≥60) HRQoL in the study setting. The mean physical and cognitive functioning scores in esophageal cancer patients were 62.0 ± 1.7 and 78.0 ± 1.9, respectively. Nonetheless, the enrolled esophageal cancer patients had poor HRQoL (score on the role, emotional and social domains <60) in the role (46.5 ± 2.5), emotional (52.6 ± 2.6), and social domains (28.3 ± 2.1) of HRQoL (Table 2). The mean score of all symptoms scales of EORTC QLQ‐C30 except financial difficulties (79.4 ± 2.1) was <60. Most of the EORTC QLQ‐OES18 symptoms scales also had a mean score of less than 60. Nevertheless, the dysphagia and financial difficulties mean scores were 72.2 ± 1.7 and 79.4 ± 2.1, respectively (Table 2).

**TABLE 2:** HRQoL among patients with gastrointestinal cancer.

Questionnaire	Scale/item	Esophageal cancer patients (*n* = 160)	Gastric cancer patients (*n* = 103)	Colorectal cancer patients (*n* = 96)
		Mean ± SEM	Mean ± SEM	Mean ± SEM
EORTC QLQ‐C30	Global health status	47.0 ± 1.5	50.7 ± 1.6	48.9 ± 1.9
	**Functional scales**			
	Cognitive functioning	78.0 ± 1.9	85.4 ± 1.9	83.0 ± 2.0
	Physical functioning	62.0 ± 1.7	57.2 ± 2.1	65.9 ± 2.0
	Emotional functioning	52.6 ± 2.6	62.5 ± 3.5	52.9 ± 3.4
	Role functioning	46.5 ± 2.5	37.1 ± 3.0	58.5 ± 3.0
	Social functioning	28.3 ± 2.1	36.6. ±2.9	44.1 ± 2.8
	**Symptom scales/items**			
	Financial difficulties	79.4 ± 2.1	81.2 ± 2.7	70.1 ± 2.9
	Appetite loss	51.3 ± 2.7	50.8 ± 3.2	42.4 ± 3.6
	Fatigue	50.9 ± 2.0	53.4 ± 2.2	47.9 ± 2.7
	Pain	49.1 ± 2.4	52.1 ± 2.9	31.8 ± 2.7
	Nausea and vomiting	33.2 ± 2.4	29.9 ± 2.9	22.4 ± 2.3
	Constipation	20.3 ± 2.3	16.2 ± 2.4	13.5 ± 2.3
	Diarrhea	20.0 ± 2.3	16.5 ± 2.3	16.0 ± 2.5
	Insomnia	18.1 ± 2.0	14.9 ± 2.4	25.7 ± 3.1
	Dyspnoea	14.0 ± 1.8	18.4 ± 3.1	16.3 ± 2.7
EORTC QLQ‐ OES18	**Symptom scales/items**			
	Dysphagia	72.2 ± 1.7		
	Trouble with taste	55.8 ± 2.6		
	Reflux	47.3 ± 2.2		
	Trouble swallowing saliva	31.7 ± 2.7		
	Eating	33.1 ± 1.8		
	Dry mouth	29.2 ± 2.6		
	Trouble with coughing	24.6 ± 2.5		
	Pain	23.9 ± 1.7		
	Choked when swallowing	23.5 ± 2.3		
	Trouble talking	19.8 ± 2.0		
EORTC QLQ‐ STO22	**Symptom scales/items**			
	Taste		67.3 ± 3.3	
	Anxiety		56.2 ± 3.1	
	Reflux		55.9 ± 2.6	
	Pain		50.4 ± 2.5	
	Eating		47.7 ± 2.8	
	Body image		34.0 ± 3.5	
	Dysphagia		22.1 ± 2.2	
	Dry mouth		21.7 ± 3.1	
	Hair loss		6.8 ± 1.9	
EORTC QLQ‐CR29	**Functional scales**			
	Sexual interest (women)			92.3 ± 2.4
	Sexual interest (men)			78.1 ± 6.3
	Body image			66.8 ± 2.9
	Weight			58.0 ± 3.6
	Anxiety			41.0 ± 3.5
	**Symptom scales/items**			
	Taste			40.0 ± 3.1
	Bloating			39.2 ± 3.2
	Flatulence			34.0 ± 2.8
	Abdominal pain			31.9 ± 2.7
	Sore skin			30.6 ± 2.9
	Urinary frequency			30.2 ± 2.3
	Stool frequency			24.1 ± 1.9
	Buttock pain			21.9 ± 2.9
	Dry mouth			20.1 ± 2.8
	Blood and mucus in stool			18.2 ± 2.4
	Fecal incontinence			16.0 ± 2.5
	Embarrassment			15.3 ± 2.6
	Dysuria			13.5 ± 1.9
	Hair loss			12.8 ± 2.1
	Stoma care problems			5.6 ± 1.7
	Urinary incontinence			4.5 ± 1.2
	Impotence			3.8 ± 1.4
	Dyspareunia			1.0 ± 0.6

*Note*: EORTC QLQ 30: European Organisation for Research and Treatment of Cancer quality of life questionnaire, EORTC QLQ‐OES18: European Organisation for Research and Treatment of Cancer quality of life questionnaire for esophageal Cancer, SEM: Standard error of the mean. EORTC QLQ‐STO22: European Organisation for Research and Treatment of Cancer quality of life questionnaire for gastric Cancer. EORTC QLQ‐CR29: European Organisation for Research and Treatment of Cancer quality of life questionnaire for Colorectal Cancer.

Concerning gastric cancer patients the study depicted that 72.8% (*N* = 75) of patients had a poor (score on the global scale <60) overall HRQoL, while 27.2% (*N* = 28) had a good (score on the global scale ≥60) HRQoL. The mean scores for emotional and cognitive functioning were 62.5 ± 3.5 and 85.4 ± 1.9 among gastric cancer patients, respectively. The mean scores of the physical (57.2 ± 2.1), role (37.1 ± 3.0), and social (36.6 ± 2.9) functioning were below 60 (Table 2). In almost all of the symptom scales of EORTC QLQ‐C30 and EORTC QLQ‐STO22, the mean score was below 60 among gastric cancer patients. However, taste problems (67.3 ± 3.3) and financial difficulties (81.2 ± 2.7) were the major issues in the symptom scales of the HRQoL domain among patients with gastric cancer (Table 2).

Regarding colorectal cancer patients, most of them (*N* = 72, 75%) had a poor (score on the global scale <60) overall HRQoL, while 25% (*N* = 24) had a good (score on the global scale ≥60) overall HRQoL. As per the EORTC QLQ‐C30 scale, colorectal cancer patients had good physical (65.9 ± 2.0) and cognitive (83.0 ± 2.0) functioning HRQoL. However, colorectal cancer patients had poor role (58.5 ± 3.0), emotional (52.9 ± 3.4), and social (44.1 ± 2.8) functioning. According to the EORTC QLQ‐CR29 scale, colorectal cancer patients had good body image (66.8 ± 2.9), and sexual interest in both men (78.1 ± 6.3) and women (92.3 ± 2.4) though they had poor mean anxiety (41.0 ± 3.5) and weight score (58.0 ± 3.6). In the EORTC QLQ‐C30 and EORTC QLQ‐CR29 symptom scales, most of the symptoms had a mean score of less than 60, indicating the absence of major symptoms‐related problems (Table 2).

In the global health status, the mean difference in the quality of life score was not statistically different (*p* > .05) among all gastrointestinal cancer types. However, in the role functioning domains of quality of life, a significant mean difference was observed between esophageal and gastric cancer patients (*p* = .04), between esophageal and colorectal cancer patients (*p* = .01), and between colorectal and gastric cancer patients (*p* < .001). In the physical functioning domain, a significant mean difference (*p* = .01) was observed only between gastric and colorectal cancer patients. In the cognitive domain, a significant mean difference (*p* = .02) was observed only between esophageal and gastric cancer patients. In the social domains of quality of life, a significant difference was shown between esophageal and gastric (*p* = .04) and colorectal (*p* < .001) cancer patients. Nonetheless, the mean difference (*p* = .14) was not significant between gastric and colorectal cancer patients in social functioning (Table 3).

**TABLE 3:** Multiple comparisons of mean difference of various domains of quality of life according to cancer type.

Quality of life domain	(I) Group	(J) Group	Mean difference (I−J)	SEM	*p*‐value
Global health status	Esophageal cancer	Gastric cancer	3.7	2.3	.25
		Colorectal cancer	1.8	2.4	.72
	Colorectal cancer	Gastric cancer	1.9	2.6	.76
Physical functioning	Esophageal cancer	Gastric cancer	4.8	2.7	.17
		Colorectal cancer	3.9	2.7	.33
	Colorectal cancer	Gastric cancer	8.8	3.0	.01*
Role functioning	Esophageal cancer	Gastric cancer	9.4	3.9	.04*
		Colorectal cancer	12.1	4.0	.01*
	Colorectal cancer	Gastric cancer	21.4	4.4	<.001*
Emotional functioning	Esophageal cancer	Gastric cancer	9.9	4.3	.06
		Colorectal cancer	0.3	4.4	1.00
	Colorectal cancer	Gastric cancer	9.7	4.8	.11
Cognitive functioning	Esophageal cancer	Gastric cancer	7.4	2.7	.02*
		Colorectal cancer	5.0	2.8	.18
	Colorectal cancer	Gastric cancer	2.5	3.1	.71
Social functioning	Esophageal cancer	Gastric cancer	8.2	3.5	.04*
		Colorectal cancer	15.7	3.6	<.001*
	Colorectal cancer	Gastric cancer	7.5	4.0	.14
Fatigue	Esophageal cancer	Gastric cancer	2.4	3.2	.72
		Colorectal cancer	3.1	3.2	.61
	Colorectal cancer	Gastric cancer	5.5	3.6	.27
Nausea and vomiting	Esophageal cancer	Gastric cancer	3.3	3.6	.64
		Colorectal cancer	11	3.7	.01*
	Colorectal cancer	Gastric cancer	7.5	4.1	.16
Pain	Esophageal cancer	Gastric cancer	3.0	3.7	.69
		Colorectal cancer	17.2	3.8	<.001*
	Colorectal cancer	Gastric cancer	20.3	4.2	<.001*
Dyspnea	Esophageal cancer	Gastric cancer	4.5	3.3	.37
		Colorectal cancer	2.4	3.4	.77
	Colorectal cancer	Gastric cancer	2.1	3.8	.84
Insomnia	Esophageal cancer	Gastric cancer	3.2	3.4	.61
		Colorectal cancer	7.6	3.5	.08
	Colorectal cancer	Gastric cancer	10.8	3.8	.01*
Appetite loss	Esophageal cancer	Gastric cancer	0.4	4.3	.99
		Colorectal cancer	8.9	4.4	.11
	Colorectal cancer	Gastric cancer	8.4	4.8	.19
Constipation	Esophageal cancer	Gastric cancer	3.8	3.3	.47
		Colorectal cancer	6.5	3.3	.13
	Colorectal cancer	Gastric cancer	2.6	3.7	.75
Diarrhea	Esophageal cancer	Gastric cancer	3.5	3.4	.55
		Colorectal cancer	4.0	3.4	.47
	Colorectal cancer	Gastric cancer	0.5	3.8	.99
Financial difficulties	Esophageal cancer	Gastric cancer	1.9	3.5	.86
		Colorectal cancer	9.2	3.5	.03*
	Colorectal cancer	Gastric cancer	11.1	3.9	.01*

Abbreviation: SEM, standard error of the mean.

* The mean difference is significant at the .05 level.

### 3.3 HRQoL according to the treatment modalities

Gastrointestinal cancer patients who underwent different treatment approaches exhibited a low mean score in the global health status and a high mean score in cognitive domains of quality of life. Among all gastrointestinal cancer patients, the various treatment modalities resulted in a generally low mean score in the social functioning domains except for chemotherapy and surgery combination‐treated colorectal cancer patients (66.7 ± 2.2) (Table 4). A one‐way ANOVA post hoc analysis showed that there was no significant difference (*p* > .05) in the quality of life score among the various treatment modalities of esophageal, gastric, and colorectal cancer.

**TABLE 4:** Different domains of HRQoL scores according to the treatment modalities.

Cancer type and their regimens	Health‐related quality of life domains					
	Global health status	Physical functioning	Role functioning	Emotional functioning	Cognitive functioning	Social functioning
**Esophageal cancer**						
Surgery	45.2 ± 2.8	62.3 ± 3.3	44.9 ± 3.9	45.1 ± 4.9	76.5 ± 3.6	27.2 ± 3.9
Chemotherapy and radiotherapy	46.4 ± 3.9	58.9 ± 3.8	45.3 ± 5.7	60.2 ± 5.6	77.6 ± 4.3	29.7 ± 5.2
Chemotherapy	55.6 ± 3.2	66.7 ± 2.4	44.4 ± 1.2	43.1 ± 1.3	72.2 ± 1.5	13.9 ± 1.6
Radiotherapy	42.6 ± 3.1	59.2 ± 5.2	46.1 ± 6.9	52.5 ± 2.3	77.5 ± 5.1	33.3 ± 6.5
Surgery and chemotherapy	47.2 ± 1.2	71.1 ± 2.3	55.6 ± 2.2	38.9 ± 5.5	83.3 ± 2.6	27.8 ± 2.3
Symptomatic management	50.6 ± 2.9	63.6 ± 4.2	47.8 ± .8	63.1 ± 2.4	81.7 ± 4.4	26.7 ± 5.1
Radiotherapy, chemotherapy and surgery	45.5 ± 4.6	66.7 ± 5.3	57.6 ± 2.3	48.5 ± 2.3	75.8 ± 5.2	40.9 ± 2.4
Radiotherapy and surgery	50.7 ± 5.9	60.0 ± 2.5	41.7 ± 2.6	49.3 ± 1.3	80.6 ± 2.7	22.2 ± 2.5
**Gastric cancer**						
Chemotherapy and radiotherapy	48.2 ± 3.3	56.8 ± 3.7	37.7 ± 2.4	70.7 ± 2.2	87.7 ± 3.2	37.7 ± 2.3
Chemotherapy	52.8 ± 2.3	56.7 ± 2.2	47.2 ± 2.2	61.1 ± 2.2	86.1 ± 5.2	50.0 ± 2.1
Radiotherapy	49.5 ± 2.9	55.9 ± 4.2	33.3 ± 4.6	60.2 ± 6.4	87.0 ± 3.1	32.9 ± 4.0
Surgery and chemotherapy	41.7 ± 2.2	66.7 ± 2.1	66.7 ± 2.3	50.0 ± 2.4	100.0 ± 2.2	66.7 ± 2.2
Symptomatic management	49.4 ± 4.2	56.4 ± 5.4	25.6 ± 6.2	58.3 ± 3.7	82.1 ± 5.8	32.1 ± 2.4
Radiotherapy, chemotherapy and surgery	56.3 ± 3.9	63.0 ± 3.4	48.3 ± 3.4	60.4 ± 2.2	80.8 ± 5.4	41.7 ± 3.5
Chemotherapy and radiotherapy	52.1 ± 4.5	41.7 ± 2.3	25.0 ± 2.2	66.7 ± 2.2	87.5 ± 2.5	25.0 ± 2.4
**Colorectal cancer**						
Surgery	57.6 ± 5.2	64.4 ± 2.2	62.5 ± 2.5	67.4 ± 2.4	87.5 ± 5.1	44.4 ± 5.9
Chemotherapy and radiotherapy	52.6 ± 5.1	63.8 ± 4.5	51.1 ± 2.4	61.9 ± 2.4	79.2 ± 5.7	44.8 ± 5.6
Chemotherapy	44.4 ± 4.1	69.5 ± 3.3	61.9 ± 6.7	53.2 ± 2.4	83.3 ± 4.3	55.6 ± 6.8
Radiotherapy	58.3 ± 1.2	73.3 ± 2.3	50.0 ± 2.4	25.0 ± 2.5	100.0 ± 2.3	16.7 ± 3.4
Surgery and chemotherapy	49.0 ± 3.1	66.9 ± 3.4	62.7 ± 5.3	52.0 ± 6.3	86.0 ± 3.6	42.7 ± 5.6
Symptomatic management	43.1 ± 1.2	65.6 ± 6.9	55.6 ± 1.4	45.8 ± 1.5	72.2 ± 1.1	36.1 ± 3.1
Radiotherapy, chemotherapy and surgery	45.6 ± 5.5	62.2 ± 5.8	53.3 ± 2.3	37.2 ± 2.5	81.1 ± 5.1	34.4 ± 6.1

A substantial proportion of esophageal cancer patients who underwent esophagectomy (*N* = 36, 22.5%) had poor overall HRQoL. In addition, 15% (*N* = 24) of esophageal cancer patients treated with a combination of chemotherapy and radiotherapy also had poor overall HRQoL. In gastric cancer patients, chemotherapy (*N* = 18, 17.5%) and gastrectomy (*N* = 28, 27.2%) treated patients had a significant deranged HRQoL. Similarly, chemotherapy (*N* = 18, 18.8%) and a combination of surgery and chemotherapy (*N* = 21, 21.9%) treated colorectal cancer patients had a significantly reduced HRQoL. However, radiotherapy (*N* = 1, 1%) treated gastric and colorectal cancer patients had minimally deranged HRQoL (Table 5).

**TABLE 5:** HRQoL according to the treatment modalities.

	Good HRQoL (mean score ≥ 60) frequency, *N* (%)	Poor HRQoL (mean score < 60) frequency, *N* (%)
Esophageal cancer (*n* = 160)		
Esophagectomy	13 (8.1)	36 (22.5)
Chemotherapy and radiotherapy	8 (5.0)	24 (15.0)
Chemotherapy	2 (1.3)	4 (2.5)
Radiotherapy	1 (0.6)	16 (10.0)
Esophagectomy and chemotherapy	1 (0.6)	2 (1.3)
Symptomatic management	10 (6.3)	20 (12.5)
Radiotherapy, chemotherapy and esophagectomy	2 (1.3)	9 (5.6)
Radiotherapy and esophagectomy	5 (3.1)	7 (4.3)
Gastric cancer (*n* = 103)		
Gastrectomy	5 (4.9)	18 (17.5)
Chemotherapy and radiotherapy	2 (1.9)	4 (3.9)
Chemotherapy	8 (7.8)	28 (27.2)
Radiotherapy	0 (0.0)	1 (1.0)
Gastrectomy and chemotherapy	3 (2.9)	10 (9.7)
Symptomatic management	9 (8.7)	11 (10.7)
Radiotherapy, chemotherapy and gastrectomy	1 (1.0)	3 (2.9)
Colorectal cancer (*n* = 96)		
Surgery	5 (5.2)	7 (7.3)
Chemotherapy and radiotherapy	7 (7.3)	9 (9.4)
Chemotherapy	3 (3.1)	18 (18.8)
Radiotherapy	0 (0.0)	1 (1.0)
Surgery and chemotherapy	4 (4.2)	21 (21.9)
Symptomatic management	1 (1.0)	5 (5.2)
Radiotherapy, chemotherapy and surgery	4 (4.2)	11 (11.5)

Abbreviation: HRQoL, health‐related quality of life.

### 3.4 Determinants of HRQoL among gastrointestinal cancer patients

Esophageal cancer patients with co‐morbidity were 3.9 times (AOR = 3.9, 95% CI = 2.4–5.8, *p* = .02) more likely to have poor HRQoL compared to patients without co‐morbidities. In gastric cancer patients, co‐morbid patients had 2.3 times (AOR = 2.3, 95% CI = 2.2–4.6, *p* = .01) more likely to have a poor HRQoL than patients without co‐morbid conditions. Likewise, co‐morbid colorectal cancer patients had higher odds of worse HRQoL (AOR = 2.5, 95% CI = 1.3–4.5, *p* = .03). Furthermore, advanced‐stage (Stages III and IV) esophageal (AOR = 2.8, 95% CI = 1.3–3.7, *p* = .03), gastric (AOR = 1.8, 95% CI = 1.5–5.3, *p* = .04) and colorectal (AOR = 10.3, 95% CI = 1.8–13.4, *p* = .03) cancer patients had a higher odds of having a poor HRQoL as compared to patients with early‐stage disease (Stages I and II). The age, gender, education level, histological type, and treatment regimens were not significant determinants of poor HRQoL (Table 6).

**TABLE 6:** Determinants of HRQoL among gastrointestinal cancer patients.

	Esophageal cancer				Gastric cancer				Colorectal cancer			
Variable	Bivariate analysis		Multivariate analysis		Bivariate analysis		Multivariate analysis		Bivariate analysis		Multivariate analysis	
	COR (95% CI)	*p*‐value	AOR (95% CI)	*p*‐value	COR (95% CI)	*p*‐value	AOR (95% CI)	*p*‐value	COR (95% CI)	*p*‐value	AOR (95% CI)	*p*‐value
Age (in years)												
<60 years	1		1		1		1		1		1	
≥60 years	1.1 (0.5–2.2)	.8	1.2 (0.5–2.6)	.7	1.3 (0.5–3)	.6	1.7 (0.6–4.6)	.3	1.5 (0.6–3.8)	.4	0.9 (0.3–3.1)	.9
Gender												
Male	1		1		1		1		1		1	
Female	0.8 (0.4–1.7)	.6	1.1 (0.5–2.4)	.8	1.1 (0.4–2.6)	.8	1.1 (0.4–3)	.8	1.4 (0.6–3.7)	.5	1.5 (0.5–4.2)	.4
Education level												
Formal education	1		1		1		1		1		1	
Informal education	1.6 (0.6–4.6)	.4	1.8 (0.6–5.9)	.3	0.3 (0.1–1)	.06	0.2 (0.1–2.2)	.1	1.2 (0.2–6.7)	.8	1.5 (0.2–10.8)	.7
Co‐morbidity												
Absent			1		1		1		1		1	
Present	2.8 (1.4–2.7)	**.03** *	3.9 (2.4–5.8)	**.02** *	2.5 (1.2–4.2)	**.04** *	2.3 (2.2–4.6)	**.01** *	1.5 (1.2–2.4)	**.02** *	2.5 (1.3–4.5)	**.03** *
Histological type												
Squamous cell carcinoma	1		1		1		1		1		1	
Adenocarcinoma	0.4 (0.1–1.9)	.2	0.4 (0.1–1.8)	.2	0.6 (0.2–1.4)	.4	0.5 (0.3–1.2)	.2	0.6 (0.2–1.4)	.7	0.7 (0.3–1.6)	.8
Stage of the disease												
Early stage (I andII)	1		1		1		1		1		1	
Advanced stage (III and IV)	1.7 (1.4–2.5)	**.04** *	2.8 (1.3–3.7)	**.03** *	2.8 (1.4–4.1)	**.02** *	1.8 (1.5–5.3)	**.04** *	2.3 (1.3–4.9)	**.03** *	10.3 (1.8–13.4)	**.03** *
Treatment regimen												
Combination therapy	1		1		1		1		1		1	
Surgery	0.8 (0.4–1.9)	.7	0.9 (0.4–2.1)	.8	1.9 (0.2–1.2)	.6	2.4 (0.7–8.6)	.2	0.4 (0.1–1.3)	.1	0.1 (0.1–0.9)	.4
Chemotherapy	1.2 (0.2–6.9)	.8	1.5 (0.2–9.6)	.7	1.8 (0.4–1.3)	.7	1.5 (0.5–4.5)	.5	1.9 (0.5–7.6)	.3	2.2 (0.5–8.9)	.3
Radiotherapy	0.1 (0.2–1.2)	.1	0.1 (0.2–1.2)	.06	1.2 (0.2–0.6)	.2	1.2 (0.3–1.4)	1.0	1.7 (0.6–2.3)	.2	0.1 (0.3–2.2)	1.0

Abbreviations: AOR, adjusted odds ratio; CI, confidence interval; COR, crude odds ratio.

* Statistically significant, *p*‐value ≤.05 (in bold).

## 4 DISCUSSION

Although several studies indicated a generally diminished HRQoL in gastrointestinal cancer patients,^10^, ^19^, ^20^, ^21^ there is a notable scarcity of research focusing on HRQoL in gastrointestinal cancer patients in sub‐Saharan Africa, including Kenya. Therefore, this study purposed to investigate HRQoL among esophageal, gastric, and colorectal cancer patients.

The study revealed that esophageal cancer patients had poor overall HRQoL which suggests the need to ensure effective treatment and improve long‐term outcomes to enhance quality of life. This finding is in agreement with other studies which reported a significantly impaired HRQoL among esophageal cancer patients.^21^, ^30^, ^31^, ^32^, ^33^ Various studies reported that older, co‐morbid, and advanced‐stage cancer patients had poor HRQoL.^15^, ^34^, ^35^, ^36^, ^37^ Hence, this high burden of poor HRQoL revealed in our study could be linked to the predominance of co‐morbid and advanced‐stage esophageal cancer patients in our setting. In sub‐Saharan Africa, cancer care is suboptimal due to the shortage of diagnostic facilities and the high cost of treatment.^38^ Therefore, this lack of access to optimal healthcare services and treatments can also worsen the low HRQoL.

The mean HRQoL score of physical and cognitive functioning was higher in esophageal cancer patients, suggesting good HRQoL in these domains. In contrast, studies in Sweden revealed that esophageal cancer patients had poor HRQoL in their physical functioning.^30^, ^39^ However, esophageal cancer patients had suboptimal HRQoL in the role, emotional, global health, and social domains of HRQoL that might be related to psychological distress due to the diagnosis of cancer and its treatment‐related adverse drug reactions and future uncertainties.

The majority of esophageal cancer patients had good HRQoL in the symptom scales, except for challenges related to financial difficulties. In contrast, several studies showed poor HRQoL in the symptom scales among esophageal cancer patients.^30^, ^39^, ^40^ These variations could be the possibility of having better symptomatic management care in our setting as a national referral facility.

The majority of gastric cancer patients exhibited poor overall HRQoL. This is in agreement with other studies.^16^, ^20^, ^41^ The mean emotional and cognitive functioning scores were higher among gastric cancer patients. However, the mean score of the physical, role, global health, and social functioning was low (<60), suggesting a poor HRQoL in those functional scales of HRQoL in gastric cancer patients. Similarly, previous studies reported gastric cancer patients had a worse functioning score in most domains.^30^, ^42^ Therefore, optimal management and early initiation treatment modalities are essential to improve this domain of HRQoL. In the symptom domains, most gastric cancer patients had a good quality of life except for the problem with taste symptoms.

Colorectal cancer patients generally exhibited a suboptimal score (<60) on the global, role, emotional, and social functioning even though physical and cognitive functioning were satisfactory. This finding is contrasted with the German study which reported a high median score in all the physical domains and global scales.^43^ Moreover, previous studies reported that most colorectal cancer patients had good HRQoL in the global score.^44^, ^45^ These disparities in HRQoL between our setting and other studies are likely attributable to differences in the quality of care, stage of disease, and co‐morbidity. The higher prevalence of co‐morbidities^46^ and the advanced stages of diseases^47^ at diagnosis may be linked to the poor HRQoL in the above domains due to the refractory nature of the diseases and the complexity of regimens used to treat those conditions.

In the symptom scale, colorectal cancer patients had a mean score of less than 60 in most of the symptom items, indicating the absence of major symptoms‐related problems. Vietnamese and Chinese studies reported substantial problems with pain and anxiety symptoms among colorectal cancer patients.^17^, ^48^ In addition, most colorectal cancer survivors had long‐term depression, distress, and bowel problems.^45^ The absence of major symptoms‐related problems in colorectal cancer patients might be related to the availability of effective symptom management and social support in the study setting. Furthermore, studies have documented that a significant number of cancer survivors face financial difficulties,^49^, ^50^, ^51^ suggesting that a subsidized cost of cancer care may be vital in improving HRQoL in colorectal cancer patients.

The study showed that there was no significant difference (*p* > .05) in the global HRQoL among different types of gastrointestinal cancers. Additionally, no statistically significant differences (*p* > .05) were observed in HRQoL domains across various treatment modalities of gastrointestinal cancer. In the social domain of HRQoL, esophageal cancer patients had the lowest mean score as compared to gastric (*p* = .04) and colorectal (*p* < .001) cancer patients. In the cognitive domain, esophageal cancer patients had also a significantly lower mean score (*p* = .02) than gastric cancer patients. Moreover, colorectal cancer patients had a higher mean score in physical functioning (*p* = .01) as compared to gastric cancer patients. Nonetheless, gastric cancer patients had the highest mean score in emotional functioning domains of quality of life as compared to esophageal (*p* = .04) and colorectal (*p* < .001) cancer patients.

In our setting, co‐morbidities and advanced stage of disease were the significant determinants of poor HRQoL. This is probably due to the necessity of more extensive and aggressive treatment regimens which can derange HRQoL. A Chinese study revealed that the level of education and nutritional support significantly affected the HRQoL in esophageal cancer patients.^31^ A study showed that patients with early‐stage disease had a better HRQoL than advanced‐stage esophageal cancer patients.^52^ An Ethiopian review reported the metastatic stage and low income level as determinants of poor HRQoL in cancer patients.^53^ Hence, it is crucial to implement vigilant monitoring and promptly initiate the most effective treatment approaches for patients with comorbidities and advanced‐stage gastrointestinal cancer.

### 4.1 Strengths and limitations of the study

The study comprehensively investigated HRQoL by using standard general and cancer‐specific HRQoL assessment tools among the selected gastrointestinal cancers. This was the first study that investigated the determinants of HRQoL in esophageal, gastric, and colorectal cancer patients in Kenya. Hence, it can be used as baseline data for further studies. Nonetheless, the study was conducted in a single healthcare facility and did not address the long‐term impacts of various treatment approaches on HRQoL. Moreover, the tools used to assess HRQoL require the patients to recall events that happened in the past. Thus, the responses were dependent on the individuals' memories, and recall bias was possible. Because of the cross‐sectional nature of the study, the HRQOL assessment took place only at a specific point in the patient's life, with no subsequent observations or follow‐up.

## 5 CONCLUSIONS

There was generally poor overall HRQoL as a result of advanced stages of disease at presentation and comorbid illnesses. Therefore, intensification of routine monitoring of the disease and the treatments should be actively implemented to improve the HRQoL. In our context, most patients have financial difficulties, which can contribute to a lack of optimal cancer care. This can significantly compromise the HRQoL of the patients. Therefore, healthcare institutions should provide subsidized cancer care nationwide to improve HRQoL substantially. A large prospective cohort study should be conducted to assess the long‐term impacts of various treatment modalities on the HRQoL of gastrointestinal cancer patients.

## AUTHOR CONTRIBUTIONS

**Amsalu Degu:** Conceptualization (equal); data curation (equal); formal analysis (equal); funding acquisition (equal); investigation (equal); methodology (equal); project administration (equal); resources (equal); software (equal); supervision (equal); validation (equal); visualization (equal); writing – original draft (equal); writing – review and editing (equal). **Peter N. Karimi:** Conceptualization (equal); data curation (equal); formal analysis (equal); funding acquisition (equal); investigation (equal); methodology (equal); project administration (equal); resources (equal); software (equal); supervision (equal); validation (equal); visualization (equal); writing – original draft (equal); writing – review and editing (equal). **Sylvia A. Opanga:** Conceptualization (equal); data curation (equal); formal analysis (equal); funding acquisition (equal); investigation (equal); methodology (equal); project administration (equal); resources (equal); software (equal); supervision (equal); validation (equal); visualization (equal); writing – original draft (equal); writing – review and editing (equal). **David G. Nyamu:** Conceptualization (equal); data curation (equal); formal analysis (equal); funding acquisition (equal); investigation (equal); methodology (equal); project administration (equal); resources (equal); software (equal); supervision (equal); validation (equal); visualization (equal); writing – original draft (equal); writing – review and editing (equal).

## FUNDING INFORMATION

The research was carried out with funding provided by the United States International University–Africa.

## CONFLICT OF INTEREST STATEMENT

The authors declare no conflict of interest.

## ETHICS STATEMENT

The study received approval from the Ethics and Research Committee of Kenyatta National Hospital/University of Nairobi (Approval No: KNH‐ERC/A/337). To safeguard the patients' privacy, their identification and residential information were not documented during the data collection process. After getting written consent from the patients, the interview was conducted in a private room of the hospital to ensure the privacy and confidentiality of patients. Official permission was also obtained from the European Organization for Research and Treatment of Cancer to use the validated HRQoL questionnaire for the respective cancer types.

## PATIENT CONSENT STATEMENT

Before data collection, we secured written informed consent from all participants in the study.

## Data Availability

The electronic version of the data will be acquired from the corresponding author.
